# Follicular Dendritic Cell Sarcoma in a Patient With Adolescent-Onset Crohn’s Disease Exposed to Multiple Immunomodulator and Biologic Therapies

**DOI:** 10.1097/PG9.0000000000000231

**Published:** 2022-07-18

**Authors:** Kaitlyn Anderson, Kerry Moss, Brendan Campbell, Douglas Moote, Kari Kakazu, Jeffrey S. Hyams

**Affiliations:** From the * Department of Graduate Medical Education, Connecticut Children’s Medical Center, The University of Connecticut School of Medicine, Hartford, CT; † Department of Hematology/Oncology, Connecticut Children’s Medical Center, The University of Connecticut School of Medicine, Hartford, CT; ‡ Department of Surgery, Connecticut Children’s Medical Center, The University of Connecticut School of Medicine, Hartford, CT; § Department of Radiology, Connecticut Children’s Medical Center, The University of Connecticut School of Medicine, Hartford, CT; ∥ Department of Pathology, Connecticut Children’s Medical Center, The University of Connecticut School of Medicine, Hartford, CT; ¶ Department of Digestive Diseases, Hepatology, and Nutrition, Connecticut Children’s Medical Center, The University of Connecticut School of Medicine, Hartford, CT.

**Keywords:** inflammatory bowel disease, infliximab, methotrexate, vedolizumab, sarcoma

## Abstract

Children and adolescents with inflammatory bowel disease are often treated with immunomodulators (thiopurines, methotrexate) and biologics (anti-TNF, anti-integrin) for extended periods despite concerns about long-term safety. Here, we report a case of follicular dendritic cell sarcoma, a very rare malignancy, and the first reported presentation in a patient with inflammatory bowel disease exposed to infliximab, methotrexate, and vedolizumab. We review the key clinical features and diagnostic factors of this malignancy. The pathogenesis of follicular dendritic cell sarcoma is largely unknown, however, knock out of B-cell TNF in mice has been related to follicular dendritic cell dysregulation through its impact on NF-κB pathways and CXCL13 chemokines. It is unknown whether any relationship exists between this patient’s diagnosis of Crohn’s disease and therapeutic exposures to this rare malignancy. We document this case in the literature to raise awareness among other clinicians who may observe a similar case.

## INTRODUCTION

Immunomodulators and biologics are considered standard of care for children and adolescents presenting with moderate to severe inflammatory bowel disease (IBD). These therapies are often introduced at diagnosis or shortly thereafter, and thus extended periods of exposure are common. Considerable experience has been published on the efficacy of anti-TNF agents (1) and anti-integrin therapy (2) in children. In this setting, however, concern persists about long-term safety, particularly with thiopurines and anti-TNF agents (3).

A large meta-analysis (4) and a large prospective study (5) examining long-term outcomes found no significant increased risk of lymphoma or other malignancies for pediatric patients on anti-TNF therapy. Less is known about the risks of therapy with newer, more targeted biologics, including gut-selective vedolizumab, particularly in the setting of previous anti-TNF and immunomodulator therapy. Early data reflect fewer and less serious adverse events (3). Here, we report a case of follicular dendritic cell sarcoma (FDCS), a rare malignancy, and to our knowledge, the first reported presentation in a patient with IBD who had exposure to infliximab, methotrexate, and vedolizumab.

## CASE REPORT

**FIGURE 1.**
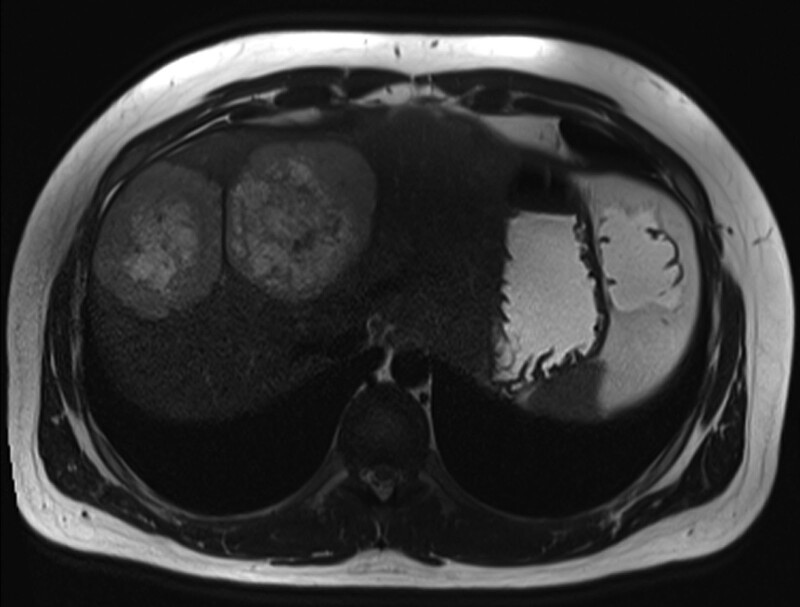
T2-weighted axial haste magnetic resonance imaging sequence demonstrates 2 right lobe liver lesions with target sign. Periphery of lesions (viable tumor) is relatively hypointense compared with center (liquefactive necrosis).

**FIGURE 2.**
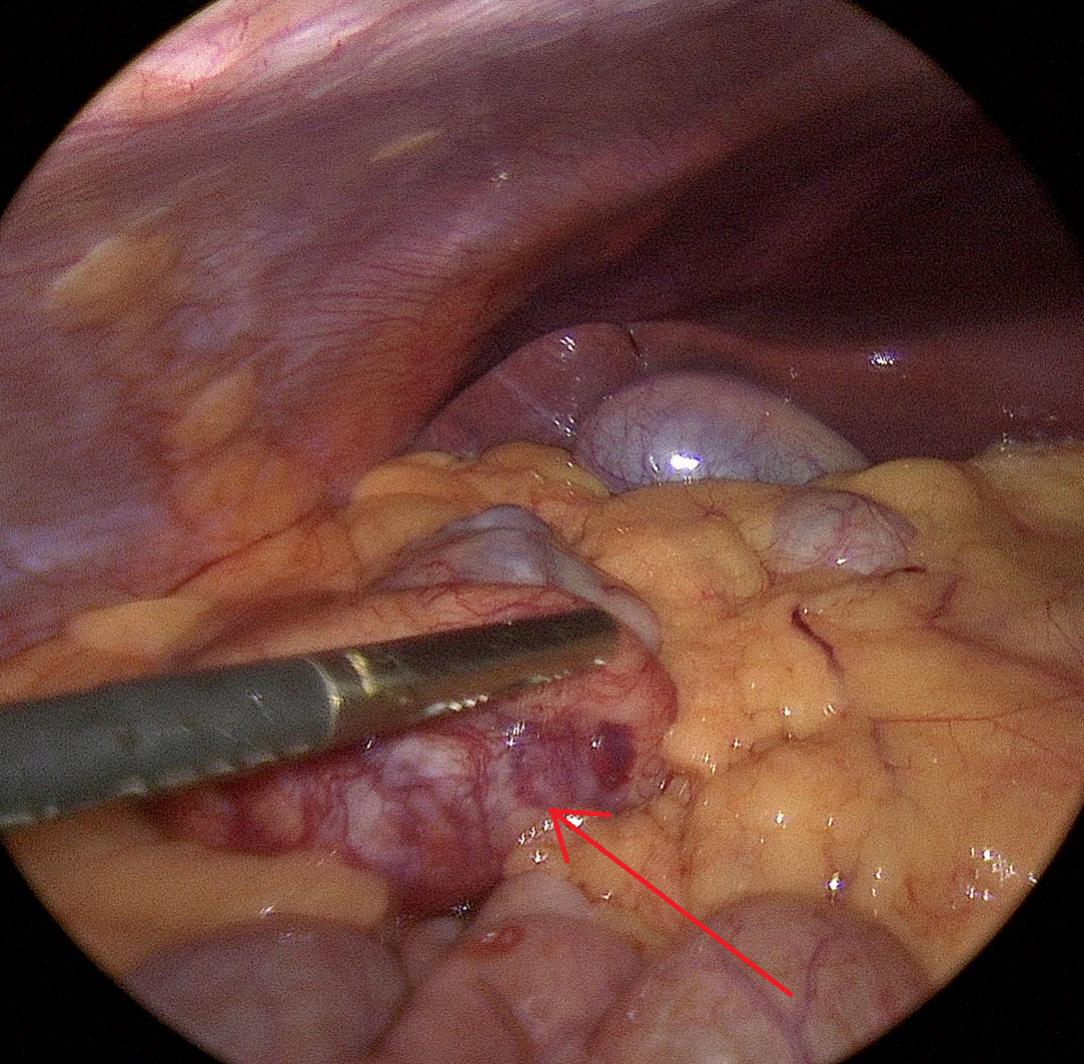
Laparoscopic view of the right upper quadrant of the abdomen from the umbilicus. The mass lies just below the laparoscopic grasper inferior to the right lobe of the liver, gallbladder, and transverse colon.

**FIGURE 3.**
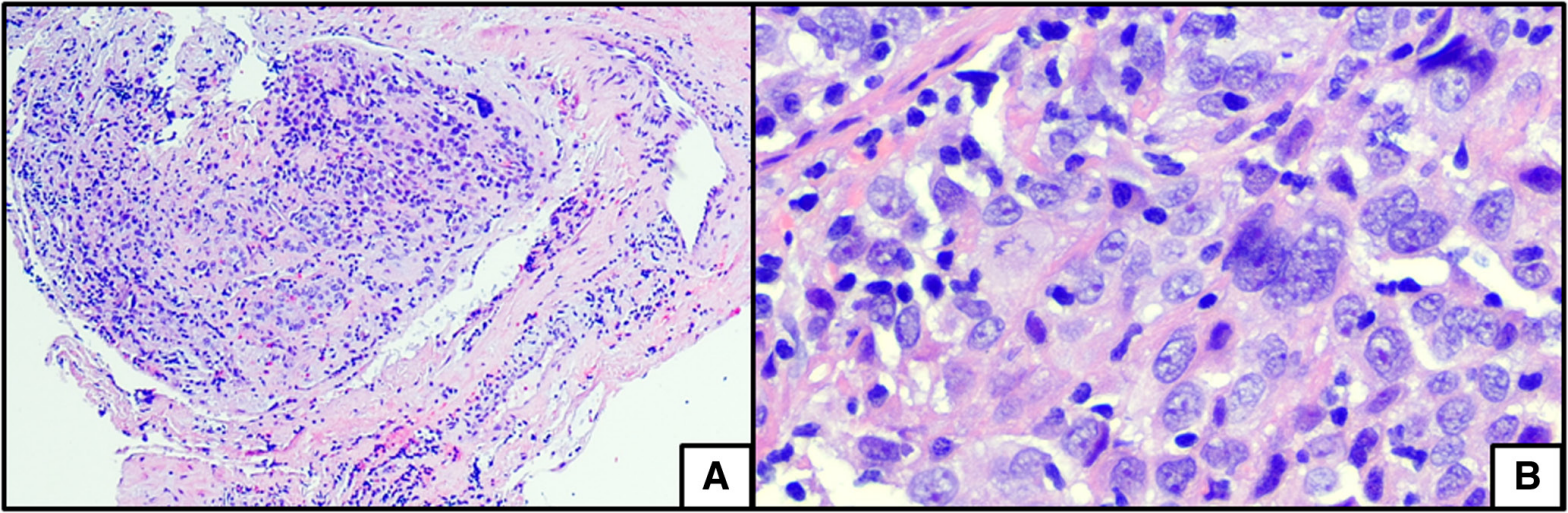
Histologic sections of the abdominal mass showing an atypical epithelioid malignant neoplasm, forming vague nodules with focally whorled bundles, associated with a relatively sparse admixture of small lymphocytes (A, hematoxylin and eosin [H&E], ×10). Neoplastic cells are large and pleomorphic with ovoid nuclei, finely granular nuclear chromatin, a few variably prominent nucleoli, and abundant pale-to-eosinophilic cytoplasm, often with indistinct cellular border (**B**, H&E, ×400).

A 21-year-old male with a history of moderately severe ileocolonic Crohn’s disease diagnosed at age 16 presented with 1 day of severe right-sided sharp abdominal pain which was unlike the pain associated with his IBD flares. Magnetic resonance enterography revealed a 5.5 × 4.1 cm mass in the mesentery of the right upper quadrant and multiple seminecrotic lesions within the liver, measuring up to 7 cm (Fig. 1). The study showed no evidence of active inflammation of the small or large bowel. Laparoscopic biopsy of the mesenteric mass (Fig. 2) revealed a markedly atypical epithelioid malignant neoplasm, with whorled bundles of large, pleomorphic cells with pale-to-eosinophilic cytoplasm, ovoid nuclei, and high mitotic activity (Fig. 3A,B). In addition to characteristic histologic features, by immunohistochemistry the neoplastic cells were positive for CD21, CD23, and CD35, diagnostic for FDCS. The tumor was negative for clusterin and was not tested for CXCL13. Staging proceeded with a computerized tomography (CT) scan of the chest, revealing right axillary lymphadenopathy and no evidence of pulmonary metastases. Positron emission tomography scan identified abnormally FDG-avid lymph nodes in the right inguinal and right axillary regions.

The patient’s medical therapy for Crohn’s disease included a short initial course of prednisone and mesalamine followed by 1 year of infliximab and methotrexate. Despite the infliximab and methotrexate, repeated flares of disease activity continued, and a follow up colonoscopy approximately 1 year after diagnosis revealed active colonic inflammation. Infliximab was stopped, and vedolizumab was started while continuing low dose methotrexate. With dosing of 300 mg every 5 weeks, the patient achieved clinical remission. Repeat ileocolonoscopy performed 13 months after starting vedolizumab was normal grossly and histologically. Total infliximab exposure was 12 months, total methotrexate exposure was 49 months, and total vedolizimab exposure was 37 months. The patient had been off infliximab for 36 months at the time of diagnosis of the malignancy.

## DISCUSSION

FDCS is a rare malignancy, accounting for <0.4% of soft tissue sarcomas, with only several hundred cases reported (6). It is characterized as an intermediate-grade sarcoma of mesenchymal-derived dendritic cells in B-follicles (6,7). The median age of presentation is 50, with a wide range of cases from young children to the elderly (7) and no gender predilection (7,8).

FDCS has been associated with the lymphoproliferative disorder, Castleman Disease, and autoimmune conditions such as pemphigus and myasthenia gravis (7,8). Jain et al, in analyzing 66 cases, found 20% to have a concurrent autoimmune disease (9). No other risk factors are known, and the pathogenesis is largely unclear. FDCS appears to be sporadic without known driver mutations, however cases are reported with identified structural alterations in tumor suppressor genes PTEN and TP53 (9).

The presenting location varies; more recent, larger studies highlight greater incidence of extranodal versus nodal disease (7,9,10). Common nodal sites include cervical, mediastinal, axillary, and intra-abdominal, while extranodal sites most often include the liver, lung, spleen, or GI tract (7,10). FDCS typically presents as a slow-growing, painless mass with no systemic symptoms; however, fever, weight loss, and abdominal pain are common features of intra-abdominal presentations (7,8). Classical FDCS can have aggressive clinical behavior with local recurrence of 50%, often with metastases (7).

The prognosis depends on the extent of disease, tumor size (<5–6 cm), surgical resectability, and histopathologic features (7,10). Poor prognostic factors include abdominal involvement, nuclear atypia, ≥5 mitoses per 10 high-power fields, lack of lymphoplasmacytic infiltration, and age <40 at diagnosis (7,10), all of which are featured in our case.

Classic histologic features include spindle-shaped epithelioid cells with a weakly eosinophilic cytoplasm in a whorled pattern, ovoid nucleus, nuclear polymorphism, low mitotic activity, and lymphoplasmacytic infiltration (7,8,10). Diagnostic immunohistochemical features include the triad of CD21, CD23, and CD35, while clusterin, CXCL13 and other markers may also be present (8,11).

We can relate aspects of the normal function and the dysregulation of follicular dendritic cells (FDC) to mechanisms of key immunomodulators used to treat IBD. FDC are important for B-cell adaptive immunity (6). B-cell TNF plays an important role in the development and maintenance of FDC, particularly in spleen, lymph nodes, and Peyers patches (12). The homeostatic chemokine CXCL13, a FDCS tumor marker, is produced by FDC and regulated by TNF (11,12). Production of CXCL13 is maintained in transformation of FDC to FDCS and is the primary driver of the lymphocytic invasion often seen in these tumor cells (11). Knockout of B-cell TNF in mice resulted in dysregulation of chemokines, such as CXCL13, which can lead to this improper infiltration of lymphocytes (12). These data also suggest that certain FDC-specific genes, which are TNF dependent, may encode CXCL13. The expression of CXCL13 genes, among other chemokines, is regulated by NF-κB (12). The expression of CXCL13 is thus dependent on TNF activation of NF-κB pathways (12). Additionally, Griffin et al (13) performed a study of targeted sequencing of a panel of several hundred known cancer-associated genes and identified recurrent loss of function alterations in tumor suppressor genes that help regulate activation of NF-κB in 38% of 13 cases of FDCS.

It is unknown whether this patient’s diagnosis of Crohn’s disease and associated therapeutic exposures are related to this rare malignancy. It is important, nonetheless, to document these cases for other clinicians who might observe a similar case.

## ACKNOWLEDGEMENT

The patient provided consent for publication of the details of this case.

## References

[1] Hyams J Damaraju L Blank M; T72 Study Group. Induction and maintenance therapy with infliximab for children with moderate to severe ulcerative colitis. Clin Gastroenterol Hepatol. 2012;10:391–9.e1. 22155755 10.1016/j.cgh.2011.11.026

[2] Singh N Rabizadeh S Jossen J. Multi-center experience of vedolizumab effectiveness in pediatric inflammatory bowel disease. Inflamm Bowel Dis. 2016;22:2121–2126. 27542130 10.1097/MIB.0000000000000865

[3] Holmer A Singh S. Overall and comparative safety of biologic and immunosuppressive therapy in inflammatory bowel diseases. Expert Rev Clin Immunol. 2019;15:969–979. 31322018 10.1080/1744666X.2019.1646127 PMC6813772

[4] Dulai PS Thompson KD Blunt HB. Risks of serious infection or lymphoma with anti-tumor necrosis factor therapy for pediatric inflammatory bowel disease: a systematic review. Clin Gastroenterol Hepatol. 2014;12:1443–51;quiz e88. 24462626 10.1016/j.cgh.2014.01.021

[5] Hyams JS Dubinsky MC Baldassano RN. Infliximab is not associated with increased risk of malignancy or hemophagocytic lymphohistiocytosis in pediatric patients with inflammatory bowel disease. Gastroenterology. 2017;152:1901–1914.e3. 28193515 10.1053/j.gastro.2017.02.004

[6] Perkins SM Shinohara ET. Interdigitating and follicular dendritic cell sarcomas: a SEER analysis. Am J Clin Oncol. 2013;36:395–398. 22772431 10.1097/COC.0b013e31824be22b

[7] Saygin C Uzunaslan D Ozguroglu M. Dendritic cell sarcoma: a pooled analysis including 462 cases with presentation of our case series. Crit Rev Oncol Hematol. 2013;88:253–271. 23755890 10.1016/j.critrevonc.2013.05.006

[8] Chen T Gopal P. Follicular dendritic cell sarcoma. Arch Pathol Lab Med. 2017;141:596–599. 28353378 10.5858/arpa.2016-0126-RS

[9] Jain P Milgrom SA Patel KP. Characteristics, management, and outcomes of patients with follicular dendritic cell sarcoma. Br J Haematol. 2017;178:403–412. 28382648 10.1111/bjh.14672 PMC5903684

[10] Hassan U Rana IA Mushtaq S. Follicular dendritic cell sarcoma of gastrointestinal tract: an uncommon lesion, commonly missed. J Gastrointest Cancer. 2019;50:913–918. 30430359 10.1007/s12029-018-0178-0

[11] Vermi W Lonardi S Bosisio D. Identification of CXCL13 as a new marker for follicular dendritic cell sarcoma. J Pathol. 2008;216:356–364. 18792075 10.1002/path.2420

[12] Tumanov AV Grivennikov SI Kruglov AA. Cellular source and molecular form of TNF specify its distinct functions in organization of secondary lymphoid organs. Blood. 2010;116:3456–3464. 20634375 10.1182/blood-2009-10-249177 PMC3321833

[13] Griffin GK Sholl LM Lindeman NI. Targeted genomic sequencing of follicular dendritic cell sarcoma reveals recurrent alterations in NF-κB regulatory genes. Mod Pathol. 2016;29:67–74. 26564005 10.1038/modpathol.2015.130

